# Applications of Fluorescence Spectroscopy, RGB- and MultiSpectral Imaging for Quality Determinations of White Meat: A Review

**DOI:** 10.3390/bios12020076

**Published:** 2022-01-28

**Authors:** Ke-Jun Fan, Wen-Hao Su

**Affiliations:** College of Engineering, China Agricultural University, Beijing 100083, China; fankejun@cau.edu.cn

**Keywords:** white meat, multispectral imaging, fluorescence spectroscopy, convolutional neural network, quality detection

## Abstract

Fluorescence spectroscopy, color imaging and multispectral imaging (MSI) have emerged as effective analytical methods for the non-destructive detection of quality attributes of various white meat products such as fish, shrimp, chicken, duck and goose. Based on machine learning and convolutional neural network, these techniques can not only be used to determine the freshness and category of white meat through imaging and analysis, but can also be used to detect various harmful substances in meat products to prevent stale and spoiled meat from entering the market and causing harm to consumer health and even the ecosystem. The development of quality inspection systems based on such techniques to measure and classify white meat quality parameters will help improve the productivity and economic efficiency of the meat industry, as well as the health of consumers. Herein, a comprehensive review and discussion of the literature on fluorescence spectroscopy, color imaging and MSI is presented. The principles of these three techniques, the quality analysis models selected and the research results of non-destructive determinations of white meat quality over the last decade or so are analyzed and summarized. The review is conducted in this highly practical research field in order to provide information for future research directions. The conclusions detail how these efficient and convenient imaging and analytical techniques can be used for non-destructive quality evaluation of white meat in the laboratory and in industry.

## 1. Introduction

As a global issue, food safety and quality are of increasing concern to companies and customers [1]. White meat is the nutritional term for lighter-colored meat that contains less myoglobin than red meat, which contains a great deal. Compared with white meat, the intake of red meat has a greater correlation with colorectal cancer (CRC), indicating that white meat intake is more beneficial to human health [2]. White meat includes poultry (e.g., chicken, duck, goose and turkey), fish, reptiles (e.g., land snail), amphibians (e.g., frog), crustaceans (e.g., shrimp and crab) and bivalves (e.g., oyster and clam), but it excludes all mammal flesh such as beef, pork, and lamb. White meat has high nutritional value and plays an important role in human diet. The production and sale of white meat need to meet specific quality and safety standards. The freshness of fish is one of the important indicators for evaluating its quality because of its high perishability [3]. Moreover, poultry products are particularly susceptible to oxidation as this meat contains relatively high levels of unsaturated fatty acids and low levels of natural antioxidants, such as vitamin E. In addition, chemical residues in white meat may have an adverse effect on human health. For example, fluoroquinolone antibiotics are effective against a wide range of Gram-negative and positive bacteria, thus they are widely used in the medical and veterinary fields. However, their use in animals has raised concerns, as this practice may lead to an increase in microbial resistance [4]. Moreover, nitrofuran drugs (NFs), including furazolidone (FZD), nitrofurazone (NFZ), and furantazone (FTD) are broad-spectrum antimicrobials. The potential risk of these compounds to human health is of great concern because of their carcinogenic and mutagenic properties. It is therefore crucial to ensure the quality and safety of white meat.

Traditional methods for meat quality and safety evaluation, such as manual inspection, mechanical and chemical methods, are time-consuming and destructive, and cannot meet the requirements of rapid inspection [5]. For example, methods for freshness evaluation are based on human sensory qualities, such as appearance, taste and texture. However, human senses exhibit a very high degree of subjectivity and can therefore be questioned in certain situations [3]. Even if manual inspection could meet accuracy requirements, it is still a labor-intensive and time-consuming process. Recently, the meat industry has adopted the most advanced high-speed processing technologies, and meat processors need fast, non-destructive, easy-to-use techniques to control the safety and quality of meat and meat products in order to achieve economic benefits. The requirement for real-time monitoring of food has encouraged the development of non-destructive measurement systems [6]. Optical technology is becoming increasingly important in research and industrial applications to measure the quality attributes of meat and meat products in real time, non-destructively and accurately [7]. Among these, the use of neural network-based RGB imaging technology has become very popular in recent years [8]. In addition, fluorescence spectroscopy and multispectral imaging (MSI) also show obvious advantages and capabilities in the non-destructive evaluation of white meat.

There have been several reviews of these new techniques of meat quality assessment. These papers show that these spectroscopic methods have been implemented as an alternative to traditional methods, but they mainly focus on one technique for quality detection of one specific category of meat, e.g., fish [3], shrimp [4], chicken [9], duck [10], or red meat [11]. As far as we know, there is no literature review analyzing the application of various imaging techniques in the non-destructive quality inspection of various white meats. (The published reviews based on these three imaging techniques are tabulated in Table 1). Considering the importance of white meat, there is an urgent need for a systematic presentation of the recent applications of spectroscopic methods to white meat. Furthermore, the published reviews cover only one aspect of meat quality measurement and lack a comprehensive review of the application of three key aspects: freshness testing, detection of harmful substances and species identification. Therefore, it appears important to review the application of the three techniques based on fluorescence spectroscopy, RGB imaging and MSI in white meat quality determination. The advantages, disadvantages and highlights of these techniques are analyzed and evaluated. This will provide a future direction for white meat quality evaluation and point out research trends for these techniques.

## 2. Fluorescence Spectroscopy, RGB- and Multispectral-Imaging

**Figure 1:**
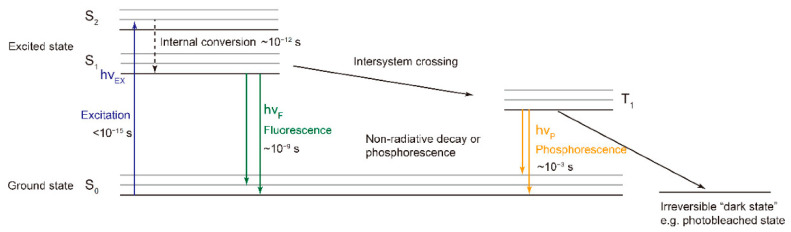
Jablonski diagram of the electron energy levels and transitions of fluorophores [29].

Fluorescence spectroscopy has proven to be an effective analytical technique over the last decade for monitoring the properties of various food products [27]. The number of published papers and citations on the use of fluorescence spectroscopy to study food quality and/or authenticity has increased exponentially over the last decade. Fluorescence is the emission of light by a fluorophore following the absorption of ultraviolet or visible light [28]. Fluorophores absorb energy as light at specific wavelengths and release energy as light at higher wavelengths. The Jablonski diagram in Figure 1 illustrates the electron energy levels of fluorophores, with the jumps between them indicated by arrows [29]. Fluorescent compounds are highly sensitive to their environment, so fluorescence can be used to characterize the conformational changes that occur under different production and storage conditions [21]. For specific applications, fluorescence analysis has the lowest background levels, low detection limits and is readily available in most laboratories [30]. 

**Figure 2:**
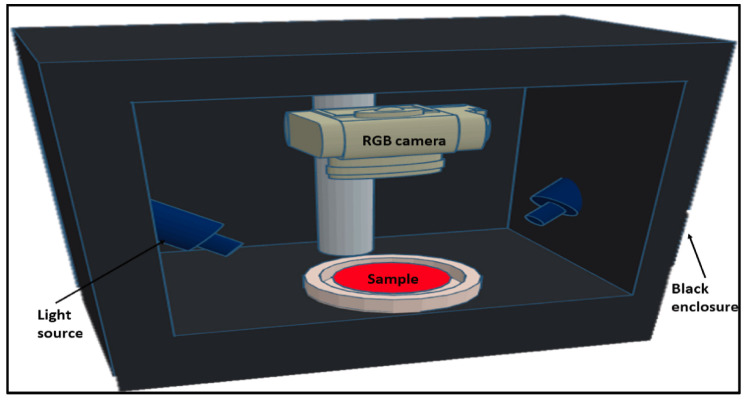
Diagram of the RGB vision system used to obtain color images of pure and contaminated meat samples [33].

RGB imaging or color imaging has gained popularity due to its clear color rendering principle, simple hardware structure and mature production process. RGB images are captured by digital cameras, webcams, or scanners from computer vision systems. These systems, typically containing an illumination system, camera and image analysis software using a computer [31], are capable of retrieving color information from captured images in the form of pixel ribbons of RGB [32]. Figure 2, for example, shows an RGB vision system for capturing color images of pure and adulterated meat samples [33]. RGB imaging has been shown to determine the general color and visual appearance of samples [34]. This imaging technology is valuable in the meat industry because it is simple, low cost and non-destructive. However, even though RGB imaging has many advantages, it only provides spatial information at a limited number of wavelengths. Conventional RGB imaging systems can be poor at identifying sensitive surface features in wavelengths other than RGB [35]. Data obtained from pure RGB imaging has been shown to be inferior to data obtained through spectral imaging when analyzing the quality of ground meat. 

**Figure 3:**
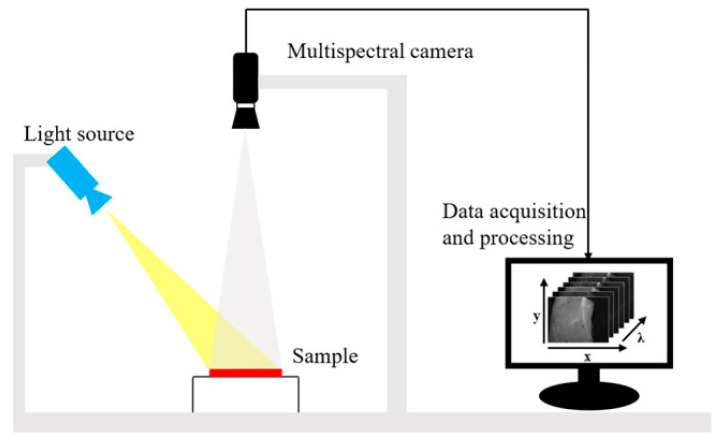
The MSI system consists of a light source (HL-2000-FHSA; Ocean Optics, Dunedin, FL, USA) and focusable lens (Nikon, Tokyo, Japan) plus a multi-channel spectral camera (miniCAM5; QHY-CCD, China) [38].

A multispectral image is a collection of grey-scale images. Each corresponds to a specific wavelength or band of wavelengths in the electromagnetic spectrum [36]. MSI is a method of capturing images from different spectral bands with the aim of obtaining spatial and spectral information. Imagers based on MSI technology can provide wavelength channels in the near-UV, visible, near-IR, mid-IR and far-IR [37]. Thus, MSI can provide more information than RGB images. The acquired wavelength channels can be used directly for real-time applications in certain fields (e.g., fruit packing plants and food processing plants). A typical MSI system is shown in Figure 3. The system uses an adjustable focus lens to achieve high resolution imaging of 1290 × 960 pixels and has six bands, each covering a relatively wide range of wavelengths, which is strong for fast imaging [38]. 

## 3. Quality Evaluation of White Meat

The application of fluorescence spectroscopy, RGB imaging and MSI for white meat quality inspection has been thoroughly and extensively researched as shown in Table 2. The following is a review of the latest applications of these techniques in non-destructive inspection. For MSI techniques, correlation coefficient (*R*) or coefficient of determination (*R*^2^) is an important statistical metric for assessing model fit, while root mean square error (RMSE) is considered an indicator of the sample standard deviation between measured and actual values, indicating that a well-performing model should obtain a high *R* or *R*^2^ value and a low RMSE value. There are many different judgements due to the variability and multiplicity of the techniques.

### 3.1. Fish

Fish is a very popular food in people’s daily diet. It is rich in amino acids, vitamins, and minerals (such as phosphorus, calcium, and iron). The quality of fish is mainly affected by storage conditions and the number of days after harvest. Mislabeling and substitutes for fish in the commercial market have been widely reported worldwide, revealing the consequences associated with economic losses, health concerns and even ocean depletion. Moreover, fish in aquaculture are susceptible to disease. Antimicrobial compounds can inhibit the growth of microorganisms in aquaculture production to prevent diseases, but their residues may accumulate in fish, posing potential health risks to consumers [86]. Thus, it is important to assess the freshness of, and harmful substances in, fish, and to accurately identify fish species. Traditional methods include sensory evaluation, chemical testing, physical characterization, and microbiological testing, which are slow and destructive. Therefore, it is necessary to use non-destructive methods to improve detection efficiency. 

Fluorescence spectroscopy plays a huge role in assessing the freshness of fish. The K value is a standard index for evaluating fish freshness. Liao, et al. [87] measured a series of K values of red snapper back meat and the corresponding fluorescence spectra of representative back scales over time. They plotted the uric acid fluorescence signal against a standard fish freshness indicator, the “K value”. This indicator was calculated using paper electrophoresis based on the concentration of adenosine triphosphate and its breakdown products. Results showed that the fluorescence intensity ratio of the emission peak at 420 nm to the peak at 310 nm increased linearly during storage (*R*^2^ = 0.95), which can be used as a non-destructive indicator of fish freshness. Besides red snapper, the fluorescence properties of Japanese dace (Tribolodon hakonensis) has also been investigated. Omwange et al. [46] developed a K value “standard freshness index” prediction model by extracting color components from the fluorescent images of the pupil and iris, and achieved good results with an RMSECV of 3.5% and *R*^2^ of 0.92. When studying mackerel (Trachurus japonicus), Rahman et al. [48] used three-dimensional fluorescence fingerprints (3D-FFs) to characterize the fluorophores in the fish. After obtaining 3D-FFs of frozen fish fillets, changes in freshness were tracked by measuring AEC values and nicotinamide adenine dinucleotide (NAD and NADH) content. Using eight and five excitation wavelengths, *R*^2^ was 0.90 and 0.85, respectively. It is thus clear that this method can be used as an effective technique for online monitoring of frozen fish quality. Later, Lai, et al. [88] developed more sensitive nano-thick fluorescent films for rapid evaluation of biogenic amines. Based on an optimized nanomembrane sensor, the detection limit for trimethylamine (TMA) was 0.89 ppm, thus enabling non-destructive evaluation of fish freshness. Overall, fluorescence spectroscopy has great potential for non-destructive assessment of fish freshness.

MSI is another effective technology for fish quality evaluation. In the study of Khoshnoudi-Nia and Moosavi-Nasab [39], MSI (430–1010 nm) combined with linear and non-linear regression methods has been used to evaluate fish spoilage. Indicators include Total-Volatile Basic Nitrogen (TVB-N) and Psychotropic Plate Count (PPC) and sensory score of the fillets. Based on nine optimal bands selected by the genetic algorithm, the non-linear models showed higher performance. Nevertheless, deep learning methods combined with other new variable selection methods should be investigated in the future. In another study, Herath, et al. [89] developed a deep neural network-based classifier using nine spectral bands for quality detection of yellowfin tuna, yielding an accuracy of 90%. The changes in docosahexaenoic acid (DHA) and eicosapentaenoic acid (EPA) in grass carp and salmon fillets were successfully determined based on acid pulp networks (PN) and genetic algorithms. Later, Dissing et al. [43] proposed a method for rapid estimation of astaxanthin concentrations in rainbow trout fillets. They used fast MSI equipment to image rainbow trout fillets and perform quantitative analysis. A partial least squares regression (PLSR) model was calibrated to predict the astaxanthin concentration from the images and showed good results with the RMSEP of 0.27. In addition, MSI based on the back-propagational artificial neural network (BP-ANN) model showed good results for predicting circular TVB-N values [90]. Nevertheless, more work should be done on the development of generic models in a wider range of fish species. 

**Figure 4:**
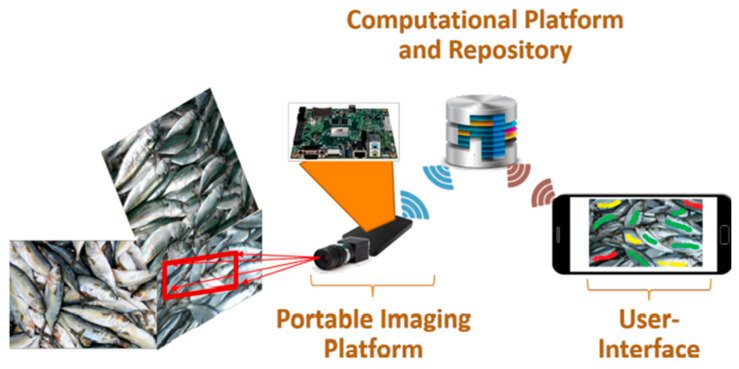
The system consists of a snapshot spectral imaging system and a mini computer system similar to the NVIDIA Jetson [3].

RGB imaging or MSI combined with convolutional neural network (CNN) can be effectively used for fish quality detection. For example, Park et al. [50] proposed an algorithm to classify fish by using CNN to train RGB images. The AlexNet-based network achieved good performance, with the shortest model training and execution time. Moreover, [3] demonstrated that the CNN model based on multispectral images showed acceptable performance in estimating the freshness of fish. A portable system was also built, as shown in Figure 4. Two CNN models established by fusing appearance and movement information successfully achieved automatic identification of the fish, with a best F score and mAP of 83.16% and 73.69%, respectively [91]. In addition, Yu, et al. [92] proposed a mask-based fish image segmentation and fish morphological feature metric scheme to pre-process and label fish images and feed them into a mask region convolutional neural network (Mask R-CNN) for training. Finally, the morphological features of fish were indexed, and the results showed that the method was able to segment fish in both pure and complex backgrounds with significant performance.

MSI has more comprehensive applications than RGB imaging and fluorescence spectroscopy in fish quality detection. Deep learning not only serves the operation of detection systems, but also allows for more efficient analysis and extraction of information. CNN, when used as a tool for analyzing images, focuses more on species identification. When combined with MSI, the results obtained are more comprehensive and convenient.

### 3.2. Crustaceans

The protein content of shrimp is as high as 20%, and its protein is at least several times higher than that of fish, eggs and milk. Freshness is considered to be a key factor for consumers in choosing shrimp, as it has an important relationship with taste and shelf life [59]. In addition, chemical residues such as fluoroquinolone antibiotics, uranium, nitrofuran metabolites, and protein arsenic were found in shrimp meat, posing a threat to human health. Therefore, it is essential to evaluate the quality of shrimp.

Fluorescence spectroscopy has been effectively used to assess the quality of shrimp. Rahman et al. [59] used multidimensional fluorescence spectroscopy to observe changes in the freshness of frozen shrimp after death. The temporal and spatial distributions of K (%) and pH in frozen shrimps were detected with prediction accuracies (*R*^2^) of 0.80 and 0.53, respectively. Fluorescence spectrometry combined with protein extraction was effectively used to analyze the protein arsenic in prawns [93]. The harmful substances in shrimp meat have also been measured. Schneider et al. [4] developed an efficient method for the multi-residue analysis of fluoroquinolones in shrimps, which allows simultaneous fluorescence quantification and multi-stage mass spectrometry confirmation. An improved programmable fluorescence detection method was then developed for determining 10 quinolones (QNs) in shrimp samples [94]. The results showed that recoveries of the 10 QNs in shrimp tissues ranged from 75.2–104.6%, with RSD values of 0.8–11.2%.

After capturing images of shrimps with RGB cameras, shrimp species recognition is usually performed using CNN. For example, Hu et al. [61] proposed a CNN-based shrimp species recognition architecture (called ShrimpNet). In ShrimpNet, two layers of CNN and two layers of fully-connected (FC) were used to obtain better shrimp recognition performance. The experimental results showed that the shrimp recognition accuracy based on the method was 95.48% in the data set of shrimp collected from six different categories, indicating that ShrimpNet has good shrimp recognition performance and practical application value. In addition, Nguyen [95] proposed a method to automatically calculate shrimp body length using an underwater camera. The CNN method obtained 87.3% mAP values with only 7% MSE values.

In summary, fluorescence spectroscopy is more commonly used than CNN-based RGB imaging techniques in the non-destructive inspection of shrimp meat quality in terms of harmful substance identification. The fluorescence detection technique mainly focuses on freshness of the shrimp meat, while CNN-based techniques are more often used for shrimp species identification and size detection for grading of shrimp meat.

### 3.3. Poultry

Poultry refers to domestic fowls, including chickens, turkeys, geese and ducks, which are mainly raised for the production of meat used as foods. Poultry is the most widely eaten meat in the world except for pork, and it provides nutritious food with high-quality protein but low fat ratio. The poultry meat should be handled properly to reduce the risk of food poisoning, but there are various potential risks (e.g., avian influenza in chickens) to the quality of meat during the rearing of poultry and the manufacture of poultry foods. In the case of chicken, for example, there are problems with sick chickens and influenza. In addition, some harmful substances in poultry meat, such as antibiotics and pesticides, can have a negative impact on human health. Therefore, it is necessary to ensure the quality of poultry. Fluorescence spectroscopy focuses on the detection of harmful substances in poultry meat. For the detection of chicken meat, Bai, et al. [96] have established the first fluorescent detection strip for chloramphenicol (CAP) residues in chicken muscle. The fluorescence intensity is detected by a charge-coupled device scanner and converted to a digital value. The performance of the test strip test was compared with that of a commercially available enzyme-linked immunosorbent assay (ELISA) kit and the *R* was 0.99, indicating the successful application of the fluorescent immunochromatographic strip for the detection of CAP residues in chicken samples.

**Figure 5:**
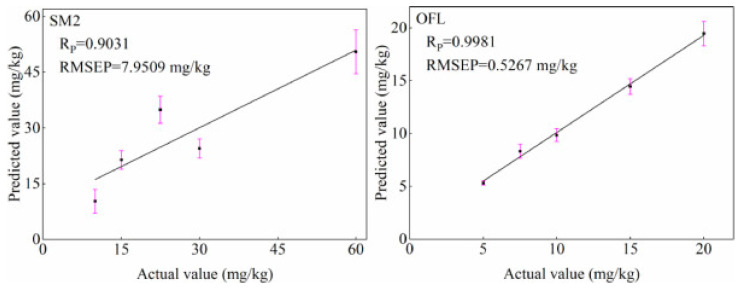
Plot of actual versus predicted values of SM2 and OFL residues in duck meat from predicted samples based on the peak height algorithm [98].

When testing goose meat, Xianglai, et al. [97] developed a method for the determination of arsenic and mercury by fluorescence spectrometry. Under the optimized operating conditions, the detection limits were 0.0048 μg/L (As) and 0.0072 μg/L (Hg), respectively. The precision was 1.91% (As) and 1.63% (Hg), respectively, indicating that the method is rapid, sensitive and accurate in the determination of hazardous substances in goose meat. In addition, a regression prediction model using the genetic algorithm combined with support vector regression (SVR) was developed for the rapid detection of carbaryl residues in duck meat using fluorescence spectrometry [10]. The results showed that the characteristic wavelengths selected by the genetic algorithm could obtain good prediction results, and the *R* and RMSEP of the predicted sample set were 0.976 and 12.232, respectively, which proved that this method could quickly detect the residues of harmful substances in duck meat. Later, for the rapid detection of antibiotic residues in duck meat, Chen, et al. [98] evaluated the potential of simultaneous fluorescence spectroscopy (SFS) combined with chemical methods for the rapid detection of sulfa-dimethoxine (SM2) and ofloxacin (OFL) residues in duck meat. A quantitative model was developed using a peak height algorithm and good results were obtained as shown in Figure 5. The method is able to meet the need for rapid detection of SM2 and OFL residues in duck meat and provides technical support for the rapid detection of antibiotic residues. Moreover, Wang, Xu, Liu, Zhao and Hong [80] developed a predictive model for the rapid detection of gentamicin residues in duck meat by fluorescence analysis based on the strong fluorescence properties of gentamicin and o-phthal-aldehyde derivatives (OPA) in the presence of emulsifier OP-10 and mercapto-ethanol. The fluorescence intensity showed a good linear relationship with the concentration of standard samples in the dynamic range 0.5~6.5 μg/mL with a linear *R* of 0.996. The *R* of the regression equation for the duck extract samples was 0.997, indicating the good performance and accuracy of the fluorescence assay in the determination of gentamicin residues in duck meat.

It is worth mentioning that the application of non-destructive quality detection based on CNN in poultry meat quality inspection is very singular and specific. Although this technique is associated less with RGB imaging, there is still a need for summary and analysis. Cuan et al. [77] proposed a new sound recognition method, the chicken sound convolutional neural network (CSCNN), for the detection of avian influenza chickens. In the experiment, the recognition accuracy of the spectrogram CSCNN-s was 93.01%, 95.05% and 97.43%, and the recognition accuracy of the feature map CSCNN-f was 89.79%, 93.56% and 95.84% on days 2, 4 and 6 after H9N2 virus injection, respectively. This indicates that the method can quickly and effectively detect chickens infected with avian influenza through chicken calls, thus preventing sick chickens from being used in chicken meat production and protecting the health of consumers. The CNN technique has played a great role in the identification of other white meat species such as fish and shrimp. However, its application in the identification and tracking of harmful substances and freshness detection is rare. In fact, as an emerging technology, the overall number of applications of CNN for non-destructive testing of white meat is still small. It is more often used as an aid for acquiring images and analyzing data to aid the operation of MSI systems. 

MSI is frequently used for the quality inspection of chicken. A simple image discrimination method for identifying chickens with systemic diseases was developed and validated across systems using two different MSI systems [9]. The first system acquired images of a batch of 164 healthy chickens and 176 systematically diseased chickens at three wavelengths of 460 nm, 540 nm and 700 nm. The verification accuracy of the healthy chickens and systematically diseased chickens was 95.7% and 97.7%, respectively. The second system acquired images of the second batch of 332 healthy chickens and 318 systematically diseased chickens at four wavelengths of 488 nm, 540 nm, 580 nm and 610 nm, and the accuracy rates were 99.7% and 93.5%, respectively. The results showed that this method can be used for automated online applications for chicken detection. In addition to this, MSI techniques are also used to detect chicken skin tumors. Spectral images of eight tumor-bearing chickens were taken in the 420–850 nm spectral range and multispectral image analysis was performed to generate graded images, which were then classified by the veterinarian as regions of interest (ROIs), as tumors or normal [65]. The image features (coefficient of variation, skewness and kurtosis) of each ROI were extracted as input to the fuzzy classifier. Using these three features, the successful detection rates were 91% and 86% for normal and tumor tissue, respectively, indicating that this method is very effective in detecting chicken skin tumors.

**Figure 6:**
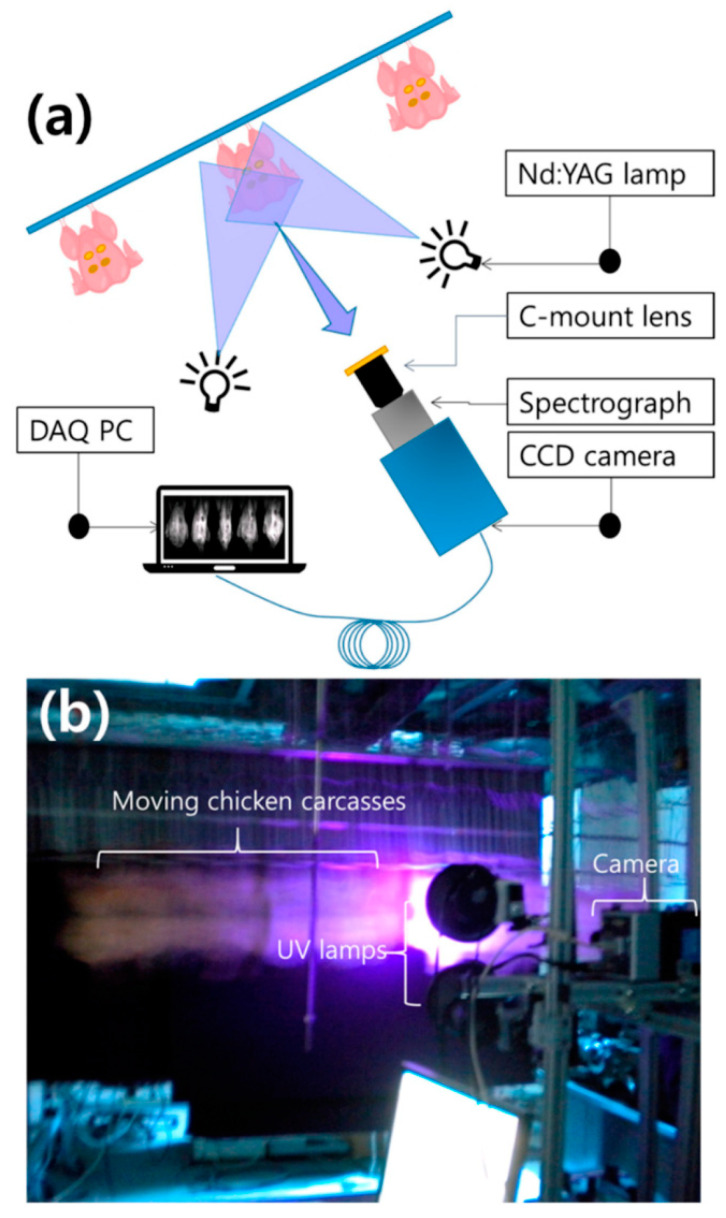
Schematic diagram of a multispectral fluorescence imaging system (**a**) and a real-time multispectral fluorescence imaging system (**b**) for the detection of fecal material on the surface of chickens [71].

Notably, Seo et al. [71] used multispectral fluorescence imaging (MFI) for the first time for online detection of poultry carcass fecal residues. As shown in Figure 6, this is a schematic and photograph of MFI. Fluorescence images were obtained by scanning four fecal spots on the skin surface of each chicken in the 410–690 nm range. The resolution between successive bands was approximately 11 nm, for a total of 27 bands. Principal component analysis (PCA) and partial least squares discriminant analysis (PLS-DA) were then performed using the spectral data from the selected areas. The results showed that both PCA and PLS-DA could distinguish areas of high and low fecal contamination from normal skin with an accuracy of 78%. However, there is a need for further research in order to develop a robust fluorescence-based detection system for various types and levels of diluted fecal contaminants.

In summary, in the current research results, fluorescence spectroscopy is more widely used for non-destructive quality inspection of poultry meat than MSI and RGB imaging techniques. While MSI focuses more on the non-destructive inspection of chicken meat, fluorescence imaging and inspection involves chicken, duck and goose meat. In addition, CNN, as a special analytical method, perform more specifically in the detection of chicken meat quality in combination with chicken calls.

### 3.4. Bivalves

Bivalvia, also known as Petromorpha Axolopoda, is the most diverse and economically valuable of the mollusk phyla, of which oysters and scallops are the types eaten regularly. For example, oysters are not only tasty and nutritious, but also have unique health and medicinal properties, making them a rare seafood product with high nutritional value. In addition, oysters have the highest zinc content of any human food. As for scallops, they are similar to fish and shrimps, and are an important aquatic food that combines food, medicine and tonicity. However, in the last few decades, industrial and urban activities have led to an increase in metal pollution, which has had a negative impact on the marine environment. Various studies have shown that marine products from industrialized coastal areas contain levels of heavy metal copper ions in excess of the standard [99]. This is the case with the meat of oysters. In addition, oysters are usually eaten fresh. They accumulate arsenic in their structure and all the arsenic species present are introduced into the human organism [100]. Aquaculture plants often dehydrate scallops to meet consumer demand. This extends its shelf life and improves the quality of the scallops. The distribution of moisture content in dried scallops is heterogeneous within individual scallops and within the same scallop, as it is influenced by the scallop and the dehydration conditions. This variability may reduce the quality of dehydrated scallops, which requires measuring of the moisture content of the dried scallops [101]. After the examples given above, it is clear that non-destructive quality determination of bivalve white meat is necessary.

**Figure 7:**
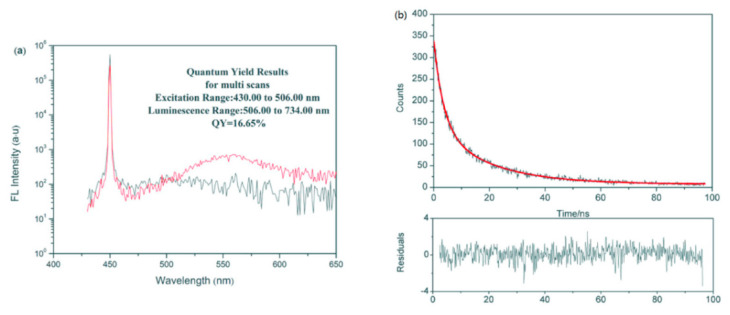
Fluorescence properties of CdSe quantum dots: (**a**) Fluorescence quantum yields of CdSe quantum dots. (**b**) Fluorescence lifetime of CdSe quantum dots [99].

As for the non-destructive testing of bivalve white meat by fluorescence detection, a method was developed for the determination of arsenic forms in oyster tissues [100]. Arsenic was measured in oysters using atomic fluorescence detection. As a result, three types of arsenic were detected in oyster tissues: arseno-betaine (AsBet) (87%), probable arsenic arsine (AsS) (4.9%) and dimethyl-arsinate (DMA) (4.7%). The method has not yet had a specific application in quality detection, but has demonstrated its ability in the detection of arsenic, a hazardous substance, and is expected to be used for non-destructive testing of white meat in oysters. Jiang et al. [99] performed a highly sensitive detection of copper ions in oysters, based on the fluorescence properties of cadmium Sinide quantum dots (Figure 7). In addition, 16 laboratories participated in a collaborative study to evaluate the performance parameters of a liquid chromatographic method for the analysis of paralytic shellfish toxins (PST) in blue mussels (*Mytilus edulis*), soft-shell clams (*Mya arenaria*), sea scallops (*Placopectin magellanicus*) and American oysters (*Crassostrea virginicus*) [102]. The method is based on reversed-phase liquid chromatography with post-column oxidation and fluorescence detection (excitation at 330 nm and emission at 390 nm). As a result of the experiments, the recoveries of individual toxins ranged from 104% to 127% and the total toxin recovery averaged 116%. Horwitz ratio (HorRat) values for individual toxins in the materials included in the study were typically in the desirable range of 0.3 to 2.0. For estimates of total toxicity in the test material, the relative standard deviation of reproducibility ranged from 4.6 to 20%.

When measuring the moisture content of dried scallops, MSI based on optimal wavelengths can be used instead of hyperspectral imaging to determine the moisture content in seafood. Huang et al. [101] used PLSR and least squares support vector machine (LSSVM) to develop a quantitative model describing the relationship between the complete spectral image and the reference moisture content value. The best wavelength combination was selected and a multispectral based image model was developed using PLSR and LSSVM modelling. Finally, the most appropriate model and visual map of the moisture content were selected. The best results, with a *R*_p_, RMSEP and residual RPD of 0.9673, 3.5584% and 3.7150 respectively, were achieved using the best wavelength-based PLSR model. These results highlight the potential of MSI for non-destructive prediction of moisture content in scallops [101]. 

## 4. Discussion

MSI has significant advantages in a number of areas when compared to other imaging techniques, such as RGB imaging and hyperspectral imaging. Compared to pure RGB imaging, MSI is the analytical tool of choice for identifying the quality of food and meat [89]. MSI has the capability to collect physical, geometric and chemical information about objects in ranges beyond the visible region. The images are produced by sensors that measure reflected energy within several spectral bands of the electromagnetic spectrum, and multispectral sensors typically measure three to ten different bands in each pixel of the image they produce for real-time applications. MSI is more widely used than RGB imaging for quality evaluation of chicken meat. In all statistical data, MSI systems have a detection accuracy of over 86%. Examples of RGB imaging systems are scarce. MSI is best suited for spectrally and spatially informative samples, and biological samples are rich in quality. As such, MSI has proven useful in a range of bioimaging applications [103]. Although hyperspectral images can provide more detail about the spectral characteristics of the object being imaged than multispectral images, the acquisition time, complexity and cost of the system are typically quite high [104,105,106,107,108,109]. Therefore, MSI using selected characteristic wavelengths is an alternative and more promising approach for the meat industry [110]. The benefits of MSI techniques are also described in more detail in the following section. Non-destructive detection of white meat can be achieved based on the fluorescence properties of specific organisms. Fluorescence spectroscopy is widely used in non-destructive quality detection of duck meat. In the data counted, the R correlation coefficients were all above 0.95. Furthermore, fluorescence spectroscopy systems are used in similar numbers compared to MSI systems with higher accuracy and correlation. Given the wide range of white meat products and the unique characteristics of some white meats, it is not possible to generalize when analyzing quality parameters. For example, freshness in chicken is usually related to the degree of lipid oxidation, while goose meat is usually judged in relation to elasticity. Fish, on the other hand, have more complex criteria depending on their type (some fish have fluorescent properties). 

CNN is often used as an algorithmic tool for analyzing data after sample images have been acquired with RGB cameras. As a deep learning method commonly used in computer vision, CNN is a class of feedforward neural networks that contains convolutional computation and has a deep structure. CNN is capable of representation learning and can perform shift-invariant classification on input information according to a hierarchical structure. Normally, CNN is more often used as a tool for analyzing images. As a technique for machine learning algorithms, it can be used for non-destructive detection of white meat. In image classification, the extraction of features from images plays a crucial role. The motivation behind CNNs is based on the extraction of attributes from automatic functions. In fact, CNNs can be thought of as automatic feature extractors. In addition to this, various CNN-based analysis techniques have emerged following the acquisition of images of various types of white meat with RGB cameras. Mask R-CNN is an algorithm that allows simultaneous target detection, target classification and instance segmentation in a neural network and is an upgraded version of Faster R-CNN [111]. Compared to Faster R-CNN, Mask R-CNN offers performance improvements in terms of time cost and accuracy. 

Neural network techniques are commonly used when analyzing multispectral images. Herath et al. [89] developed a deep neural network based fish class classifier for yellowfin tuna, and training data on the neural network achieved 90% accuracy on test data. CNN can also be used as a tool to analyze multispectral images as a means of estimating fish freshness. It is feasible to use state-of-the-art CNNs to automatically extract appropriate spatial-spectral features [3]. Hirama, et al. [112] also proposed a method to identify fish using an echogenic vocalizer connected to a set net. The proposed method uses the data obtained by CNN from echo vocalizers connected to the set net to identify fish. In the CNN-based deep learning architecture, the input is a sonar image and the output is a fish species. Using this method, five fish species were identified with 95% accuracy. In addition, a genetic algorithm was also applied to the creation of an MSI system. After imaging, the elimination of redundant bands in the spectral set is a necessary step to create simple, low cost and fast predictive models that can be used for online monitoring of food safety and quality. GAs were effectively used to select the richest wavelength variables associated with fish quality from the full spectral range [39]. In addition, the choice of an appropriate wavelength selection method can be of great help in the development of calibration models. 

In summary, fluorescence spectroscopy, RGB imaging and MSI all have advantages and disadvantages for different species of white meat. RGB imaging is more universal when fish is the object of detection, with an accuracy of over 75.00% in all cases. While fluorescence imaging is less common, it has higher accuracy and correlation coefficient values. MSI is not widely used for the detection of shrimp meat, while RGB imaging has a detection accuracy of over 90.00%. Fluorescence spectroscopy was slightly less accurate. Otherwise, the situation is even richer when the subject of testing is poultry meat. When using MSI for non-destructive testing of chicken, the number of cases detected is high but the detection accuracy is lower than the other two imaging techniques, whereas fluorescence imaging and RGB imaging showed better potential: the accuracy ranged from 94.00% to 97.43%. For duck, there is no doubt that fluorescence spectroscopy is the most powerful tool.

## 5. Challenges to Fluorescence Spectroscopy, RGB Imaging and MSI and Future Trends

By providing spectral information related to the quality characteristics of white meat, MSI techniques have proven to be an effective method for rapid non-destructive classification and detection of freshness and harmful substances in white meat. Fluorescence detection has also proven to be an effective method for tracking the level of hazardous substances in white meat by utilizing the fluorescence properties of some meats. RGB imaging is also very widely feasible for non-destructive testing of white meat. Based on these chemical-free assessment methods, the speed of non-destructive testing of white meat is greatly increased and the errors caused by subjective judgement are greatly reduced, effectively safeguarding the health of consumers. This chemical-free technique presents superior results to traditional manual testing. 

Although there is now a wealth of scientific research demonstrating the enormous capabilities and potential of these technologies for food detection, they undeniably still have various drawbacks. RGB imaging, although low cost and convenient, can only provide spatial information in a limited number of wavelengths. When using MSI for quality assessment of white meat, although efficient and complete detection is guaranteed, there is no doubt that a complete MSI system can be expensive. The main obstacle to industrial applications is budgetary constraint. In order to meet the need for cost-effectiveness, the development of an inexpensive and specific imaging system will be particularly crucial in the future. Fluorescence detection, as a long-established detection technique, does have a large number of applications in white meat quality detection. The great potential of fluorescence spectroscopy combined with multivariate statistical analysis for food quality assessment has also been demonstrated [17]. After analyzing and collating a large body of literature, we found that fluorescence spectroscopy has been used mainly for tracking harmful substances in white meat foods and, to a lesser extent, for freshness testing of meat. It is rarely used to identify the type of meat. In today’s seafood market, there are countless cases of mislabeling due to misidentification of fish species. The consequences associated with economic losses, health problems and even depletion of the oceans have become apparent. Therefore, there is still a need for laboratories to expand the use of fluorescent detection.

It is clear that these techniques, though, have an extremely high research potential for non-destructive quality evaluation of white meat. However, there is still much room for improvement and innovation when applied to industrial production and inspection. Firstly, the costs of these imaging and analytical techniques need to be controlled and reduced due to the large number of applications in the food industry. Secondly, there is a need for greater interaction and cooperation between these three techniques. Sometimes one of the three techniques will have a greater advantage when testing the quality of the same meat product. In addition, all three techniques can be used as complementary and analytical tools. This suggests that laboratories may move closer to technological interaction in the future. Finally, practical predictive models for quality parameters are also important. Even if a high degree of applicability of the technique can be guaranteed, it is also necessary to ensure that the choice of model is very sound. The two complement each other in order to effectively improve the accuracy of food quality assessment. In conclusion, based on machine learning, fluorescence spectroscopy, RGB imaging and MSI techniques are expected to be powerful tools for fast and efficient non-destructive quality detection of white meat and may lead to better intelligence, innovation and further applications in other food industries and fields.

## 6. Conclusions

In this review, the latest applications of fluorescence spectroscopy, RGB imaging and MSI are highlighted as promising techniques for non-destructive quality inspection of various white meat foods. MSI is a method of capturing images from different spectral bands, sufficient to gather physical, geometric and chemical information about objects in ranges beyond the visible region, and has proven to be the analytical tool of choice for identifying the quality of food and meat. There are more diverse methods for non-destructive quality determination of white meat using fluorescence spectroscopy. Fluorescence can be used to characterize the conformational changes that occur under different production and storage conditions and is therefore a promising process analysis tool for characterizing white meat foods. Certain organs or secretions of some fish have fluorescent properties that can also be used as indicators of fish quality. CNN techniques are able to represent learning and classification of input information based on a hierarchy of shift variants and can also be used as a tool to analyze multispectral images and RGB imaging for detecting the freshness of white meat. As a technique that does not require extensive pre-processing, it excels in the non-destructive detection of white meat and species identification. Based on the rapidly evolving excellence objectives of the modern meat industry, these three techniques have been added to the knowledge base for monitoring product quality parameters of white meat. Given the recent excellent advances and innovations in computer vision and data analysis modelling, it is expected that these techniques will not only be more intensively studied and widely used in the laboratory, but will also become extremely powerful tools for non-destructive quality assessment of white meat and other food products on an industrial scale.

## Figures and Tables

**Table 1:** Summary of reviews on fluorescence spectroscopy, RGB- and MSI techniques in food evaluation.

Technology	Product	Target Attributes	Reference
MSI	Meat	Adulteration	Ropodi et al. [12]
MSI, HSI	Meat	Defects	Feng et al. [13]
MSI	Food	Quality	Su and Sun [14]
MSI, IRS, SERS, LIBS and HSI	Food	Quality	Wang et al. [15]
MSI, HSI and VS	Food	Authenticity, quality and safety	Ropodi et al. [16]
Fluorescence spectroscopy	Food	Quality	Karoui and Blecker [17]
Fluorescence spectroscopy	Food	Quality	Strasburg and Ludescher [18]
Visible/Infrared, Raman and Fluorescence spectroscopy	Raw and processed food	Quality	He and Sun [19]
Fluorescence spectroscopy	Food	Quality	Ahmad et al. [20]
Fluorescence spectroscopy	Dairy products	Quality and safety	Shaikh and O’Donnell [21]
Fluorescence spectroscopy	Fresh and frozen-thawed muscle foods	Muscle classification	Hassoun [22]
RGB-Imaging	Meat	Quality and safety	Taheri-Garavand et al. [23]
RGB-Imaging	Fish	Quality	Dowlati et al. [24]
RGB-Imaging	Food	Quality	Gomes and Leta [25]
RGB-Imaging	Food	Quality	Amani et al. [26]

MSI––Multispectral imaging; HSI––Hyperspectral imaging; IRS––Infrared spectroscopy; SERS––Surface-Enhanced Raman Spectroscopy; LIBS––Laser induced breakdown spectroscopy; VS––Vibrational Spectroscopy.

**Table 2:** Applications of fluorescence spectroscopy, RGB imaging and MSI for quality evaluation of various white meat products.

White Meat	Module	Quality Parameters	Accuracy	Reference
Fish	MSI	TVB-N, PPC	*R*^2^_p_ = 0.862 for TVB-N, *R*^2^_p_ = 0.921 for PPC	Khoshnoudi-Nia and Moosavi-Nasab [39], Khoshnoudi-Nia and Moosavi-Nasab [40]
Fish	MSI	TVC	*R*^2^ = 0.62	Govari, et al. [41]
Fish	MSI	TVC	*R*^2^ = 0.683	Fengou, et al. [42]
Fish	MSI	Astaxanthin concentration	*R*^2^ = 0.86	Dissing, et al. [43]
Fish	MSI	TVB-N, TBARS, K	*R*^2^_p_ = 0.922 for TVB-N, *R*^2^_p_ = 0.867 for TBARS, *R*^2^_p_ = 0.936 for K	Cheng, et al. [44]
Fish	MSI	A ‘standard freshness index’ of K	*R*^2^ = 0.94,	Omwange, et al. [45]
Fish	Fluorescence spectroscopy	A ‘standard freshness index’ of K	*R*^2^ = 0.92	Omwange, et al. [46]
Fish	Fluorescence spectroscopy	A ‘standard freshness index’ of K	*R*^2^ = 0.95	Liao, et al. [47]
Fish	Fluorescence spectroscopy	AEC; NADH	*R*^2^ = 0.90 for AEC, *R*^2^ = 0.85 for NADH	Rahman, et al. [48]
Fish	Fluorescence spectroscopy	NADH	90.5%	Hassoun and Karoui [49]
Fish	RGB imaging	Classification performance	99.5%	Park, et al. [50]
Fish	RGB imaging	Astaxanthin concentration	*R*^2^ = 0.66	Dissing et al. [43]
Fish	RGB imaging	Freshness of tuna meat cuts	86.67%	Lugatiman, et al. [51]
Fish	RGB imaging	The main color of the sample	75%	Mateo, et al. [52]
Fish	RGB imaging	Texture features	86.3%	Gu, et al. [53]
Fish	RGB imaging	Color of Salmon Fillets	*R* = 0.95	Quevedo, et al. [54]
Fish	RGB imaging	Gill and eye color changes in the sparus aurata	*R*^2^ = 0.994	Dowlati, et al. [55]
Fish	RGB imaging	Body color of carp	94.97%	Taheri-Garavand, et al. [56]
Fish	RGB imaging	Freshness	98.2%	Rocculi, et al. [57]
Shrimp	Fluorescence spectroscopy	4-hexylresorcinol	81.6%	Jonker and Dekker [58]
Shrimp	Fluorescence spectroscopy	K, pH	*R*^2^ = 0.80	Rahman, et al. [59]
Shrimp	RGB imaging	pH	100%	Witjaksono, et al. [60]
Shrimp	RGB imaging	Identification accuracy of the proposed ShrimpNet for shrimp	95.48%	Hu, et al. [61]
Shrimp	RGB imaging	Shrimp dehydration levels	*R* = 0.86	Mohebbi, et al. [62]
Shrimp	RGB imaging	Color changes in the head, legs and tail of pacific white shrimp (litopenaeus vannamei)	90%	Ghasemi-Varnamkhasti, et al. [63]
Chicken	Fluorescence spectroscopy	Hydroxyproline concentration	*R*^2^ = 0.82	Monago-Maraña, et al. [64]
Chicken	MSI	Skin tumors	86%	Chao, et al. [65]
Chicken	MSI	TVC	90.4%	Spyrelli, et al. [66]
Chicken	MSI	pork-chicken adulteration	90.00% for fresh samples, 86.67% for frozen-thawed samples	Fengou, et al. [67]
Chicken	MSI	Sepsis in chickens	98.6% for septic chickens, 96.3% for healthy chickens	Yang, et al. [68]
Chicken	MSI	Contamination detection	96%	Park, et al. [69]
Chicken	MSI	Chicken heart disease characterization	100%	Chao, et al. [70]
Chicken	MSI; Fluorescence spectroscopy	Contamination detection	92.5%	Seo, et al. [71]
Chicken	Fluorescence spectroscopy	Lipid oxidation	*R* = 0.73	Gatellier, et al. [72]
Chicken	Fluorescence spectroscopy	*P. aeruginosa* concentration	96%	Abdel-Salam, et al. [73]
Chicken	Fluorescence spectroscopy	chicken meat tenderness	*R =* 0.870	Yu, et al. [74]
Chicken	Fluorescence spectroscopy	Contamination detection	96.6%	Cho, et al. [75]
Chicken	Fluorescence spectroscopy	Measurement of lipid oxidation	98%	Wold and Kvaal [76]
Chicken	RGB imaging	Avian flu infected chickens	97.43%	Cuan, et al. [77]
Chicken	RGB im-aging	Color	94%	Yumono, et al. [78]
Chicken	RGB im-aging	Freshness	*R* = 0.987	Taheri-Garavand, et al. [79]
Duck	Fluorescence spectroscopy	Gentamicin Residual in Duck Meat	*R* = 0.996	Wang, et al. [80]
Duck	Fluorescence spectroscopy	Doxycycline content in duck meat	*R* = 0.998	Wang, et al. [81]
Duck	Fluorescence spectroscopy	Carbaryl residue in duck meat	*R* = 0.976	Xiao et al. [10]
Duck	Fluorescence spectroscopy	Tetracycline content	*R* = 0.952	Zhao, et al. [82]
Duck	Fluorescence spectroscopy	Triazophos content	*R*^2^_p_ = 0.974,	Zhao, et al. [83]
Duck	Fluorescence spectroscopy	Neomycin residue	*R* = 0.999	Jiang, et al. [84]
Duck	Fluorescence spectroscopy	Carbofuran residue	*R*^2^_p_ = 0.999	XIAO, et al. [85]

TVB-N––total volatile basic nitrogen; PPC—Psycho-trophic Plate Count; TVC—total viable count; LDA—Linear Discriminant Analysis; MD—Mahalanobis distance; PCA—Principal component analysis; m—mean; TBARS—Thio-barbituric acid reactive substances; AEC—adenylate energy charge; NAD and NADH—nicotinamide adenine dinucleotide; CFU—colony-forming units; TBARS—thio-barbituric acid reactive substances.
